# Life-threatening bleeding with intussusception due to gastrointestinal stromal tumor: a case report

**DOI:** 10.1186/s40792-019-0703-9

**Published:** 2019-10-24

**Authors:** Min Sung Kim, In Teak Woo, Young Min Jo, Jin Hyung Lee, Byung Sam Park

**Affiliations:** 1 Department of Internal Medicine, Soonchunhyang University Hospital, Gumi, South Korea; 2 School of Medicine, Soonchunhyang University, Asan, South Korea; 3 Department of General Surgery, Soonchunhyang University Hospital, 179, 1gongdan-ro Gyenongsanbuk-do, Gumi, 39371 South Korea; 4 Department of Pathology, Soonchunhyang University Hospital, Gumi, South Korea

**Keywords:** Gastrointestinal stromal tumor, Intussusception, Bleeding

## Abstract

**Background:**

Massive intraluminal bleeding requires urgent intervention and management. However, the source of bleeding on the small intestine is difficult to determine. Intestinal tumor with intussusception is a rare and normally not an urgent condition. Herein, we present a rare case of intestinal intussusception with massive bleeding due to jejunal gastrointestinal stromal tumor (GIST) that required emergency surgical treatment.

**Case presentation:**

A 51-year-old male was admitted to the emergency department complaining of abdominal pain and acute hematochezia. Esophagogastroduodenoscopy (EGD) and colonoscopy could not determine the source of the bleeding site. Abdominal pelvic computed tomography (AP-CT) revealed GIST with intussusception, strongly suggestive of distal jejunal bleeding. Unresponsive transfusion with low blood pressure and continuous hematochezia led to emergency laparotomy. GIST, which was the leading point for intussusception, was located in the jejunum and showed mucosal ulceration of approximately 3.5 cm in diameter. Following resection and functional anastomosis, histology revealed a GIST with low mitotic count (< 5 per 50HPF). Moreover, immunochemical analysis revealed positivity for c-kit (CD117) and DOG-1. There were no complications 2 months after surgery.

**Conclusions:**

Intussusception associated with GIST is a rare finding that can be life-threatening if it occurs with an ulcer. This case showed that the early detection of bleeding and emergency surgery could prevent severe complications.

## Background

Massive hematochezia with melena is a fatal condition that requires an emergent intervention and possibly surgery. Usually, the origin of hematochezia is bleeding of the colorectum, small bowel, or stomach. However, bleeding of the small bowel is difficult to find and is most likely to be confirmed late [1].

Gastrointestinal stromal tumors (GISTs) are rare, representing less than 0.2% of all gastrointestinal tumors and only 0.04% of small intestinal tumors. Small bowel intussusception from GIST in adults has been described in a few cases in the literature. Most of the reported adult cases manifested in abdominal pain and obstruction symptoms, with no symptoms of massive bleeding [2–6]. Cases of GIST with massive bleeding without intussusception are rarely described in the literature [7, 8]. Herein, we present a rare case of small bowel intussusception with a massive bleeding from GIST in a 51-year-old male and discuss the diagnostic approach and surgical treatment.

## Case presentation

A 51-year-old male patient was admitted to the emergency department with massive hematochezia, hypotension, and abdominal pain. The patient reported intermittent hematochezia for 3 days. His medical history was remarkable for hypertension only. There was no history of radiation therapy or recent abdominal surgery.

**Fig. 1:**
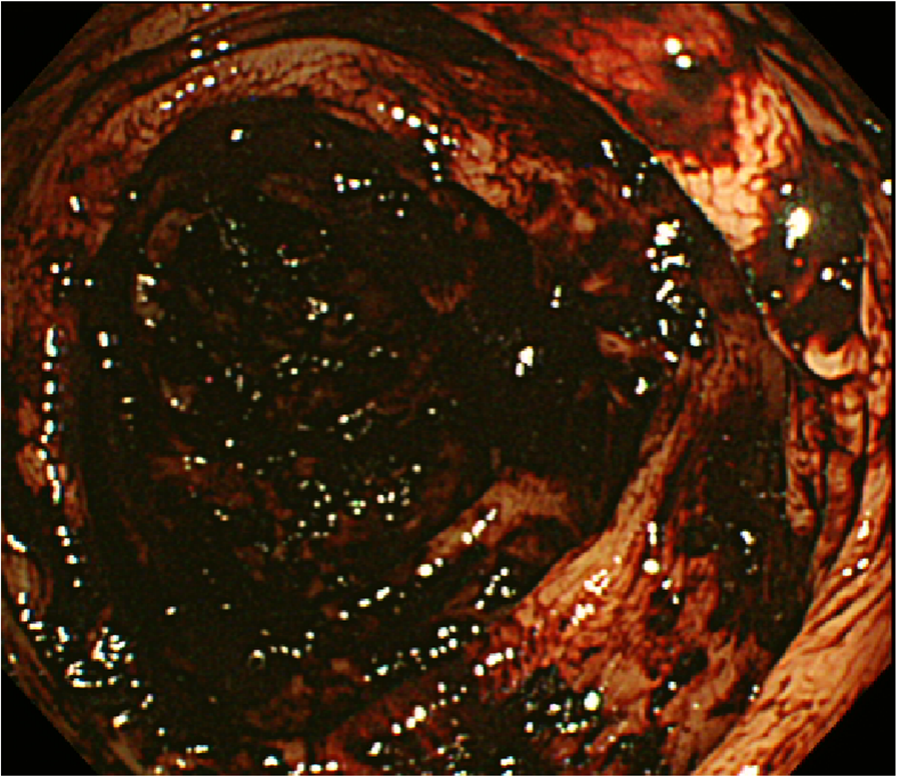
Colonoscopic finding of unknown origin melena

**Fig. 2:**
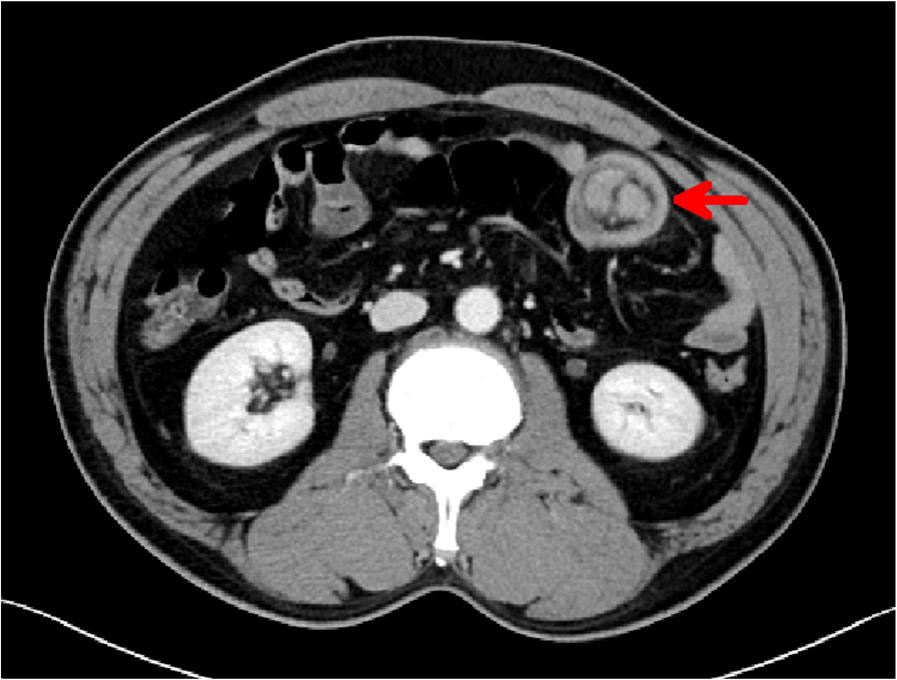
CT scan showing a target sign at the jejunum

On initial clinical observation, the patient was hemodynamically unstable. The systolic blood pressure was 90 mmHg, heart rate was 110/min, and temperature was 36.8 °C. His abdomen was soft and flat, but there was tenderness in the left upper quadrant. Because initial hemoglobin value in the emergency room was 7.3 g/dl (normal range, 13.5–17), immediate transfusion and emergency endoscopy were performed to identify bleeding sites and perform hemostasis. An esophagogastroduodenoscopy showed no upper gastrointestinal pathology to account for bleeding, and colonoscopy revealed hematic residues but no detected lesion (Fig. 1). As soon as endoscopy was completed, abdominal pelvic computed tomography (AP-CT) scan was performed with IV contrast. AP-CT scan showed that the distal jejunum and its mesentery were tortuous and the end of the mesentery was ring-shaped suggesting an intra-abdominal intussusception (Fig. 2).

**Fig. 3:**
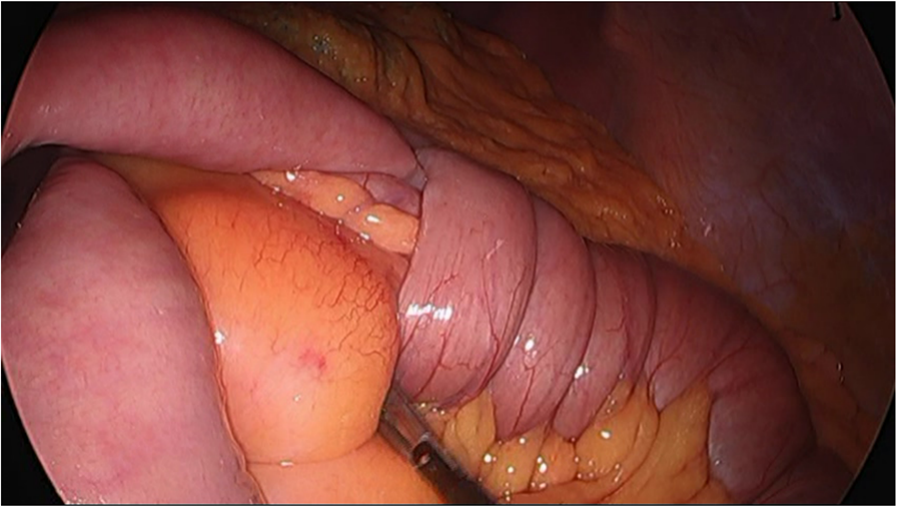
Intraoperative laparoscopic finding showed intussusception of the jejunum

Because of unresponsive transfusion for low blood pressure and continuous hematochezia, an emergency laparotomy was performed. Intraoperative laparoscopic findings showed typical intussusception in which one small bowel infiltrated the other small bowel (Fig. 3).

**Fig. 4:**
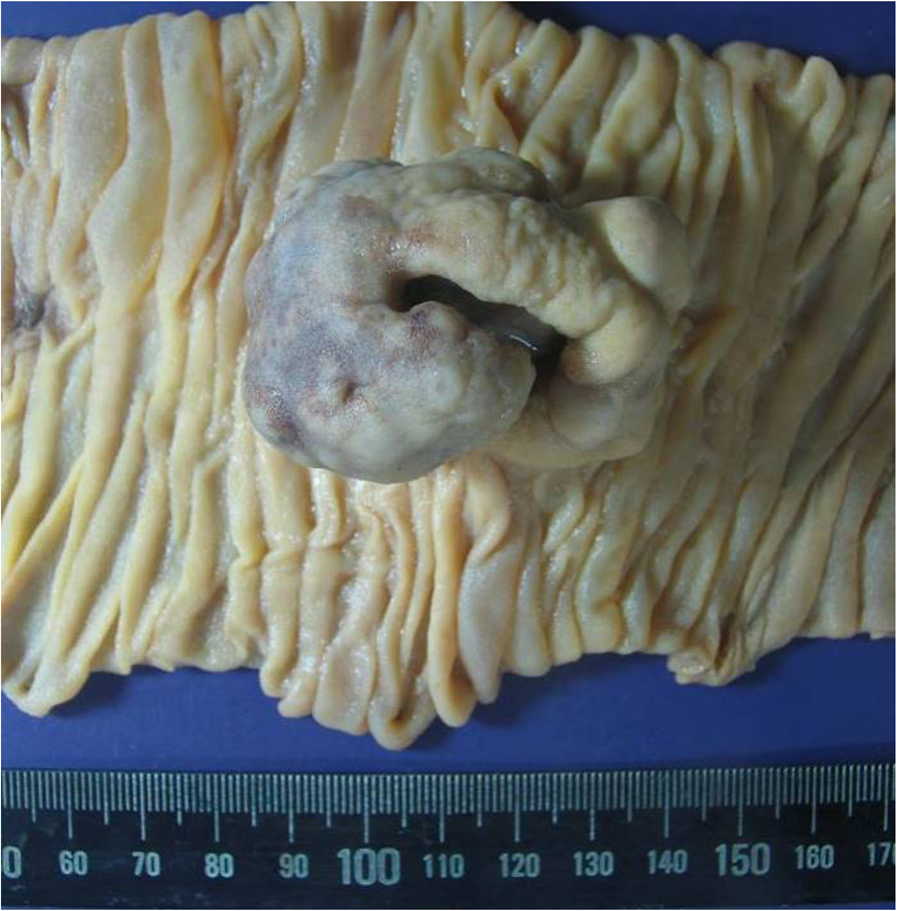
Gross imaging of gastrointestinal stromal tumor

**Fig. 5:**
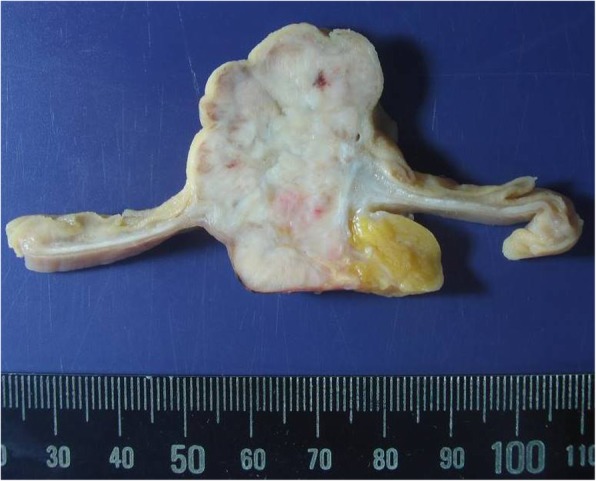
Grossly coronal resection of gastrointestinal stromal tumor

Gross and histopathologic examination after resection confirmed GIST to be the leading point causative of the intussusception. Grossly, the mass was within the small bowel wall, covered with epithelial mucosa, and measuring 3.7 × 2.5 × 2.5 cm. A centrally located and depressed ulcerative lesion was identified overlying the tumor (Fig. 4). On cut section, a relatively well-demarcated, firm and fibrotic, and light gray, lobulated mass was identified. The mass involved the entire small bowel wall, extending from the mucosa to the subserosa without penetrating the serosal surface. Overall, monotonous and multifocal hemorrhagic foci were identified, with no dominant focus of bleeding (Fig. 5).

**Fig. 6:**
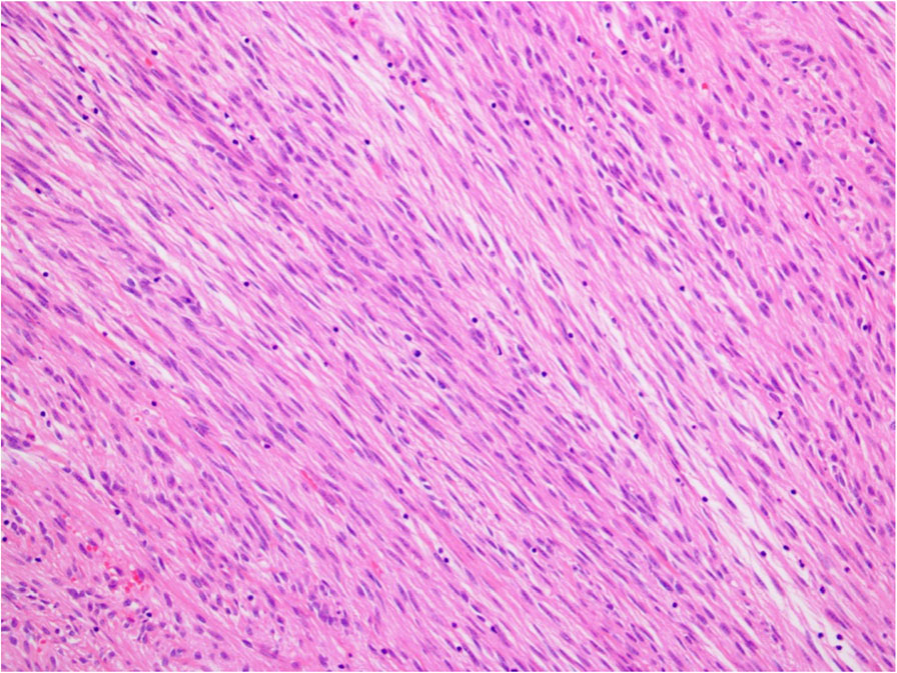
In high magnification, spindle cells showed elongated nuclei with vesicular chromatin, inconspicuous nucleoli, forming short fascicles

On light microscopic examination, hypercellular and hypocellular areas were identified with mostly hypercellular component showing nodular appearance under low magnification. Under high magnification, spindle cells showed elongated nuclei with vesicular chromatin and inconspicuous nucleoli forming short fascicles (Fig. 6). The ulcerative area showed neutrophilic inflammatory exudate and extravasated erythrocytes, confirming heavy bleeding within the ulcerative focus.

**Fig. 7:**
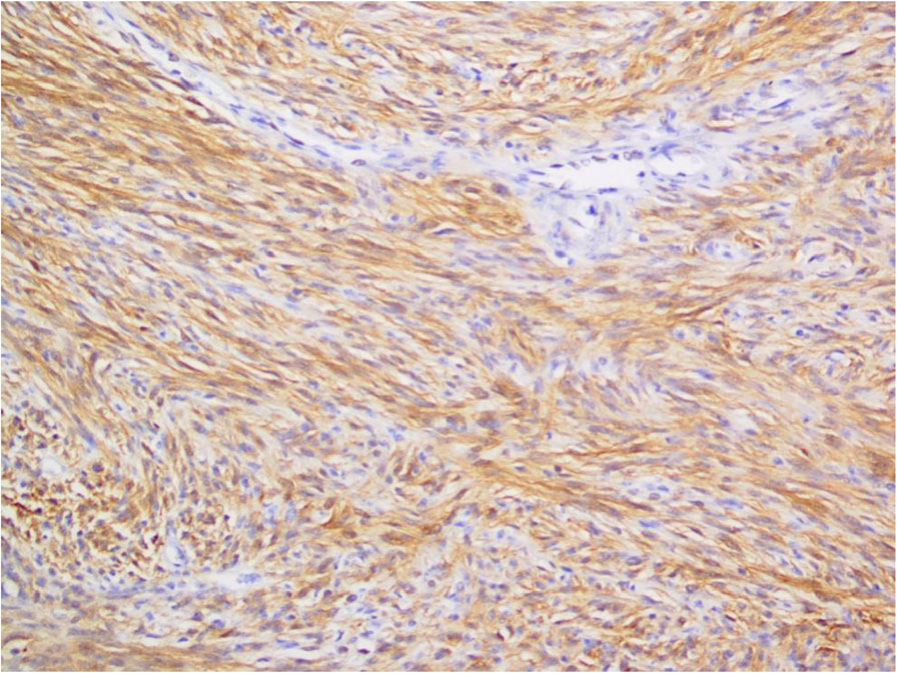
Immunohistochemically, the tumor was strongly positive for c-kit (CD117), showing diffuse and cytoplasmic reactivity

Immunohistochemically, the tumor was strongly positive for c-kit (CD117), showing diffuse and cytoplasmic reactivity (Fig. 7). DOG1 (also known as ANO1, anoctamin 1) stain was also positive in our case, which is sensitive and relatively specific for GIST, confirming the diagnosis.

## Discussion

Only 5% of all primary GI tract tumors are small bowel origin tumors [9]. It is difficult to diagnose the small bowel tumor because symptoms are often absent or non-specific. Small bowel origin GIST is a rare tumor that is also difficult to diagnose. A review of 18 cases of intussusception secondary to GIST found that approximately 39% (7/18) of GISTs were within the small bowel. None of them had massive bleeding symptoms [2–6]. Thus, our case is the first reported and extremely rare manifestation presented as small bowel intussusception with massive bleeding from GIST.

Normally, endoscopic examination is considered first in patients with hematochezia or melena symptoms to identify the bleeding focus site. But, in patients with atypical abdominal pain, such as intussusception, AP-CT can be considered as primary strategy. In this case, endoscopy was performed first because there was massive bleeding than with normal intussusception. When the endoscope failed to identify the site of bleeding, AP-CT was performed immediately. AP-CT image showed the intussusception with jejunal GIST requiring an emergency operation.

Classically, GIST is not easy to cause intussusception or bowel obstruction because it grows exogenously into the abdominal cavity and spread rarely into adjacent organs [4]. Intussusception with massive bleeding from GIST is more extremely rare, too. Also, if GISTs have a mucosal ulcer at well-developed organs of blood vessels such as the stomach, massive bleeding can occur [10]. The jejunum is a well-developed organ. In this case, GIST originated in the jejunum with accompanying intussusception and massive bleeding triggered by ulceration. Ulcers involving sites of excessive blood circulation can lead to life-threatening outcomes.

Mitotic index and tumor size are well-known parameters used to stratify GIST into low, intermediate, and malignant categories. However, the classification does not fully indicate the risk of GIST malignancy and only reflects the degree of aggressiveness. Even a small GIST with a low mitotic index may increase the risk of recurrence or metastasis to other organs and sites. Therefore, currently, GISTs are considered as malignant neoplasms, and strict criteria based on specific parameters have yet to be established [11].

However, Novitsky et al. reported that mitotic index, tumor size, tumor ulceration, patient age, and necrosis are key factors that significantly influence tumor recurrence [12]. Further, Miettinen et al. reported that the small intestinal GISTs show more aggressive features compared with the gastric GISTs of similar size and mitotic index; however, tumor ulceration has limited effect on patient’s prognosis [13, 14].

Surgical intervention is not always indicated for GIST. However, surgical resection is necessary to determine the predisposing factors. Adult intussusception is an indication for surgical resection. In this study, GISTs of small bowel origin associated with ulceration and intussusception were resected. Post-surgery, the patient was defined as a low-risk category for recurrence despite the presence of GISTs in the small intestine with ulceration. The patient was under surveillance according to the Korean guidelines for GIST [15].

## Conclusions

Life-threatening bleeding is an emergency warranting urgent intervention or surgery. We report a rare case of massive bleeding associated with intussusception involving mucosal ulceration due to GIST. Intussusception associated with critical bleeding may be triggered by GIST, which should be suspected as one of the possible causative factors. An emergency operation may be required to address the clinical signs and symptoms [13].

## Data Availability

This case report does not have a dataset. The figures supporting the conclusions of this article are included within the article.

## Abbreviations

AP-CT: Abdominal pelvic computed tomography
EGD: Esophagogastroduodenoscopy
GIST: Gastrointestinal stromal tumor
